# Carvedilol Precipitation Inhibition by the Incorporation of Polymeric Precipitation Inhibitors Using a Stable Amorphous Solid Dispersion Approach: Formulation, Characterization, and *In Vitro In Vivo* Evaluation

**DOI:** 10.3390/polym14224977

**Published:** 2022-11-17

**Authors:** Akhila Akkihebbal Ravikumar, Parthasarathi K. Kulkarni, Riyaz Ali M. Osmani, Umme Hani, Mohammed Ghazwani, Adel Al Fatease, Ali H. Alamri, Devegowda V. Gowda

**Affiliations:** 1 Department of Pharmaceutics, JSS College of Pharmacy, JSS Academy of Higher Education and Research (JSS AHER), Mysuru 570 015, Karnataka, India; 2 Department of Pharmaceutics, College of Pharmacy, King Khalid University (KKU), Guraiger, Abha 61421, Saudi Arabia; 3 Department of Pharmaceutics, Cauvery College of Pharmacy, Mysuru 570 028, Karnataka, India

**Keywords:** drug delivery, drug precipitation, amorphous solid dispersions, carvedilol, cellulose, crystallization, precipitation inhibition, solubility, supersaturation

## Abstract

An amorphous solid dispersion (ASD) of carvedilol (CVL) was prepared via the solvent evaporation method, using cellulose derivatives as polymeric precipitation inhibitors (PPIs). The prepared ASDs existed in the amorphous phase, as revealed by X-ray powder diffraction (XRPD) and scanning electron microscopy (SEM). The Fourier-transform infrared spectroscopy (FT-IR) and differential scanning calorimetry (DSC) results confirmed the compatibility between CVL and the polymers used. The ASDs characteristics were evaluated, with no change in viscosity, a pH of 6.8, a polydispersity index of 0.169, a particle size of 423–450 nm, and a zeta potential of 3.80 mV. Crystal growth inhibition was assessed for 180 min via an infusion precipitation study in simulated intestinal fluid (SIF). The interactions between the drug and polymers were established in great detail, using nuclear magnetic resonance (NMR) spectroscopy, nuclear Overhauser effect spectroscopy (NOESY), and Raman spectroscopy studies. Dielectric analysis was employed to determine the drug-polymer interactions between ion pairs and to understand ion transport behavior. In vivo oral kinetics and irritation studies performed on Wistar rats have demonstrated promising biocompatibility, stability, and the enhanced bioavailability of CVL. Collectively, the stable ASDs of CVL were developed using cellulose polymers as PPIs that would inhibit drug precipitation in the gastrointestinal tract and would aid in achieving higher in vivo drug stability and bioavailability.

## 1. Introduction

The drug discovery arena is becoming more challenging with each passing day, with a constant row of new hurdles and formulation tribulations concerning poorly water-soluble drug candidates that require new-fangled strategies for enhancing their dissolution characteristics and bioavailability [1,2]. The formulation and development of poorly water-soluble drugs remain a challenge in pharmaceutical drug discovery. In the biopharmaceutical classification system (BCS), class-II and -IV drugs exhibit poor water solubility at the intraluminal level. Thus, to enhance their dissolution, solubility, and the absorption of drugs belonging to these classes, many approaches have been adopted, including particle-size reduction, converting poorly soluble drugs into prodrug entities, creating soluble salt forms, and the use of cyclodextrins as complexing agents [3]. A particularly attractive approach in the pharmaceutical field is the conversion of crystalline drugs to an amorphous form that successfully enhances the solubility of poorly water-soluble active pharmaceutical ingredients (APIs) [4]. Amorphous drugs offer several advantages in terms of enhancing drug solubility and dissolution, while a few drugs are being formulated in a highly unstable crystalline form and are then marketed in the same form, to curtail the product development costs. However, owing to their thermodynamically unstable crystalline state, these drugs most often revert to their stable crystalline form when they come into contact with an aqueous environment [5].

Currently, many formulations are developed as solid dispersions (SDs) for poorly water-soluble drugs; these play a vital role in enhancing the rate and extent of the absorption of drugs in the gastrointestinal tract [6,7,8]. According to the Noyes–Whitney equation, a reduction in particle size leads to a significant increase in the dissolution rate of poorly water-soluble drugs; this substantially increases the bioavailability [9]. Absorption in the gastrointestinal tract is limited by the equilibrium solubility of the drug, but drug fraction permeation cannot be achieved in equilibrium solubility studies. Thus, to overcome this issue, supersaturation drug delivery offers a key answer. According to the concept of supersaturated drug delivery, poorly water-soluble drugs in higher amounts (highly dissolved and dispersed) with a high drug-free energy eventually enhance bioavailability. To achieve a stable SD formulation, maintaining supersaturation and stabilizing the amorphous form are key strategies [10].

Certain researchers have broadly studied the mechanism of solubility enhancement and the advantages of formulating drugs in an amorphous form [11,12,13]. Amorphous compounds have the advantage of increasing solubility and dissolution by reducing the energy that leads to precipitation into a crystal lattice in the supersaturated solution during the dissolution process; ultimately, this drastically reduces the drug’s bioavailability. The precipitation of a poorly water-soluble drug from a supersaturated solution involves two critical phases: nucleation and crystal growth. Higher bioavailability can be achieved in a physiologically relevant period and in the media of a supersaturated state if one or both of the phases (i.e., nucleation and crystal growth) can be inhibited. However, the recrystallization of several drugs occurs during the process of dissolution in gastrointestinal pH and during storage, leading to reduced drug bioavailability and, thereby, reduced efficacy [14,15,16,17].

Carvedilol (CVL) is a biopharmaceutics classification system (BCS) Class-II drug that is a lipid-soluble compound that is practically insoluble in water and poorly absorbed from the gastrointestinal tract, exhibiting pH-dependent solubility. It is widely used to treat a variety of cardiovascular ailments, including hypertension, heart failure, and left ventricular dysfunction following myocardial infarction. This drug is commercially available in the form of tablets for oral administration, with a low oral dose (6.25–25 mg); however, its systemic bioavailability is only 25–35%, due to extensive hepatic first-pass metabolism by cytochrome P450, and it also has a short plasma half-life. However, up to a fourfold improvement of CVL bioavailability could be achieved by increasing the CVL solubility [18,19]. Although the precipitation inhibition of drugs from aqueous supersaturated solutions using polymers has been extensively reported in the literature, drug-polymer interactions have not been widely studied [20,21,22]. To date, many studies focusing on the polymer-drug ratio have been reported [23,24,25]. In this article, a formulation based on saturated drug delivery has been discussed. Drug-polymer interactions may uphold or inhibit precipitation (nucleation and crystal growth) in an unpredictable manner, which, in turn, has a significant impact on the extent and duration of supersaturation and its bioavailability [26]. However, enhancing the solubility of poorly water-soluble drugs by using the amorphous nature of the drug in the carrier matrix to change it into a controlled release form remains a challenge. Therefore, the obtained solubility advantage can be lost upon the erratic and/or unpredictable precipitation of the drug during dissolution [27]. Poorly water-soluble drug precipitation inhibition determines the success of supersaturating the dosage form in the gastro intestinal tract (GIT) and GI milieux. However, the excipients or hydrated matrix used to obtain the amorphous state of a drug may delay or minimize release or even recrystallize the drug during storage or in the dissolution process [28]. The amorphous form of drugs can be achieved by using surfactants [29,30], polymers in the form of cellulose derivatives, such as HPMC acetate succinate [31,32], polyvinylpyrrolidone K90 [33,34], Eudragit E100 [35,36], and cyclodextrin derivatives [2,37], which can successfully inhibit the precipitation of drugs from the solution phase once the higher apparent level of drug concentration is achieved. The process of selecting the polymer is a critical factor for achieving amorphous solid dispersion; the selected polymer crafts the drug to attain an amorphous state during the drug release scenario. Cellulose polymers have been proven to exhibit more efficient and potential inhibition action on the crystallization of drugs. However, a clearer and more thorough understanding of drug-polymer interactions has yet to be established [38,39,40].

The current work aims to elucidate the effect of polymer stabilizers as an approach for inhibiting the precipitation and crystallization of drugs from supersaturated solutions of weakly basic drugs, for enhancing their oral absorption. The current work investigates the precipitation inhibition mechanism of cellulose polymers using a model drug (carvedilol) in a supersaturated state. This study also highlights current modifications and progress in the solid dispersion approach for obtaining high drug loading via the development of an efficient formulation of a drug-polymer system. For this study, the effect of individual polymers on the precipitation of the model drug in a solution phase was determined through precipitation studies, in the presence and absence of polymers. The morphological and polymorphic characterization of the collected drug precipitates was carried out to establish the parameters, to assess the precipitation inhibition extent. Nuclear magnetic resonance (NMR) spectroscopy and nuclear Overhauser effect spectroscopy (NOESY) were used to explore the interaction between the drug and polymer [41,42,43]. Raman spectroscopy studies were used to determine the drug state in the formulated solid dispersions. Furthermore, a detailed in vivo analysis of precipitation inhibition, efficiency in reducing gastric irritation, and the potential to enhance the drug bioavailability in albino Wistar rats by formulating it in solid dispersions, were evaluated and discussed in great detail.

## 2. Materials and Methods

### 2.1. Materials

The carvedilol (CVL) used in the study was a gift sample from BeloorBayir Biotech Ltd., Tumkur, India. Hydroxypropyl methylcellulose (HPMC) of different grades (E3, E6, E5, E15, E50, K4M, K100 M, and phthalate), biorelevant media (without the enzyme), NaOH, NaCl, and NaH_2_PO_4_·H_2_O were purchased from Sigma Aldrich-Merck Ltd., Mumbai, India. Ethanol and HCl were purchased from Padmashri Chemicals, Mysore, India. All the other reagents and solvents procured were of analytical grade and were used according to the manufacturer’s instructions.

### 2.2. Methods

#### 2.2.1. Inhibition Effect of Polymers on CVL Recrystallization in a Supersaturated State

The recrystallization of CVL at supersaturation was studied by dissolving CVL in 10 mL of water containing 1 mL of methanol. Furthermore, the drug solution was incorporated into various polymer solutions and was analyzed for inhibition of the crystallization process. An aliquot of CVL (in an oral dose of 3.125 µgml^−1^) was added to 900 mL of pH 6.8 fasted state simulated intestinal fluid (FaSSIF), containing 100 µgml^−1^ of polymers [44]. The pre-dissolution experiment was carried out at 37 °C at 50 rpm; the concentration of CVL was determined by the presence and absence of polymers at specified time intervals. The rate of recrystallization was calculated at 240 min, and recrystallization was determined by measuring the CVL concentration, using UV spectroscopy (UV spectrophotometer, UV-1800, Shimadzu, Kyoto, Japan).

#### 2.2.2. Preparation of a CVL Solid Dispersion (CVL-SDs)

CVL solid dispersions (CVL-SDs) were prepared by the solvent evaporation method using the vacuum evaporation technique [45]. The polymers which showed good crystallization inhibition actions were selected as “hit polymers” and formulated to achieve solid dispersion. Briefly, hydroxypropyl methylcellulose K100M and hydroxypropyl methylcellulose E15 loading dose (drug–polymer at a ratio of 1:0.75) were calculated according to the oral dose of CVL. Then, the aqueous CVL was added to the polymer, which is dissolved in an ethanol solution. At this point, the mixed solvent was subjected to evaporation under reduced pressure, using a vacuum dryer at 40 °C. Once the solvent was evaporated, the samples were dried under vacuum for at least 24 h. The pulverized powder was sieved and stored at 0% relative humidity (RH). The prepared solid dispersions were further characterized for drug–polymer interactions and drug content analysis.

#### 2.2.3. Practical Yield

The percentage of practical yield was calculated to determine the efficiency of the method adopted. Solid dispersion (SD) was collected and weighed to determine the practical yield (PY) from the equation given below:

$$Practical\;yield\;\left(\%\right)=\frac{Practical\;mass\;of\;solid\;dispersion\;}{Theoretical\;mass\;of\;polymer\;and\;drug}\times 100.$$

#### 2.2.4. Drug Content

The SD equivalent to 10 mg of CVL was weighed accurately and dissolved in 10 mL of methanol. The solution was then filtered and diluted suitably, and the drug content was analyzed at 283 nm with a UV spectrophotometer (UV-1800, Shimadzu, Kyoto, Japan). The drug content was calculated using the equation given below:

$$Drug\;content=\frac{Amount\;of\;drug\;in\;solid\;dispersion\;}{Theoretical\;amount\;of\;solid\;dispersion\;}\times 100.$$

### 2.3. Characterization of Solid Dispersion

#### 2.3.1. Powder X-ray Diffraction

Powder X-ray diffraction analyses for the pure drug and prepared ASDs were performed at room temperature, and the patterns were obtained using a Rigaku diffractometer. The diffraction patterns were obtained via Ni-filtered CuKa radiation (λ = 1.5418 Å), under a 20 mA, 40 kV voltage operation. The samples were screened in the 10- to 90-degree 2θ range, with the experimental parameters set to a scan step size of 0.020 for 2 s, with a scan speed of 0.010/s [46].

#### 2.3.2. Scanning Electron Microscopy (SEM)

The morphology and surface topography of prepared SDs were imaged with a scanning electron microscope (Zeiss EVO LS 15, Smart SEM 5.05, Jena, Germany) operating at an acceleration voltage of 15 kV and with suitable magnification, at room temperature. Briefly, the samples were mounted onto 5-mm diameter silicon wafers, followed by sputter-coating with Au under an argon atmosphere. For imaging in the SEM, specimens must be electrically conductive, at least at the surface, and electrically grounded to prevent the accumulation of an electrostatic charge at the surface. Therefore, the optimized SDs were Au-coated before being subjected to electron scanning [47].

#### 2.3.3. Atomic Force Microscopy (AFM) Analysis

The shape and surface characteristics (roughness) of SDs were analyzed by using AFM. The force acting between the probe tip of AFM and the surface of SD samples was measured by AFM (A.P.E. Research, A-100, Trieste, Italy). Moreover, the effect of SDs on delicate targets can also be studied by AFM. Depending upon the sample’s properties, AFM scanning can be performed in both contact and non-contact modes. The non-conducting samples can be easily imaged using AFM without any pretreatment. The AFM imaging of SDs was carried out in the ultra-low amplitude tapping mode, and the spatial resolution was up to 0.01 nm.

#### 2.3.4. Differential Scanning Calorimetry (DSC)

Differential scanning calorimetry analysis (DSC-60, Shimadzu, Kyoto, Japan) was carried out for the prepared SDs. For the differential analysis, high-purity alpha-alumina discs (empty cells) were adopted as the reference. High-purity indium metal was adopted as the standard for instrument calibration. Dynamic scans were performed under a nitrogen atmosphere with a heating rate of 20 °C/min, in a temperature range of 20–400 °C [48].

#### 2.3.5. Fourier-Transform Infrared Spectroscopy

FT-IR spectroscopy (FTIR-8400S, Shimadzu, Kyoto, Japan) was employed to identify the possible interaction of the drug with the polymers used. The spectra were obtained using the KBr pellet technique, within the wavelength range of 4000 cm^−1^ to 400 cm^−1^.

#### 2.3.6. Raman Spectroscopy

Raman spectral analysis is a spectroscopic technique by which the “fingerprint” of a molecule can be identified. The Raman spectrum (Xplora Plus, Horiba, Japan) was recorded by illuminating the molecule using a laser beam; spectra for the aqueous samples were recorded since water is a weak Raman scatterer.

#### 2.3.7. Dynamic Light Scattering

The products obtained in the process of formulation were further analyzed by dynamic light scattering (DLS) to determine any sub-visible aggregates. Briefly, SDs were mixed thoroughly with water for 10 min before measurement. The zeta potential of the aggregates in clear supernatant was measured by DLS (ZS Nano, Malvern Instruments Inc., Worcestershire, UK) as well as by hydrodynamic radius, then the particle size distribution was measured. The refractive index was set at 1.333. The scattered light intensity was measured at a 90° angle to the incident light. The samples were analyzed in triplicate using a clear disposable cell, with water as a dispersant at 25 °C [49].

#### 2.3.8. Transmission Electron Microscopy Analysis

The size and shape of the prepared SDs were evaluated by means of transmission electron microscopy (TEM) (Philips CM 10, Philips Electron Optics, Eindhoven, The Netherlands); the images were assembled with NIH image software. TEM analysis is based on the transmission of electron beams through ultra-thin nanoparticulate samples. Suspensions of drug-loaded or -unloaded SDs (with a 0.5% *w*/*v* concentration) were sprayed uniformly on formvar-coated copper grids, using negative materials such as phosphotungstic acid/uranyl acetate and were observed after complete air-drying. Sample fixation was carried out, in order for the samples to withstand the vacuum created by the instrument.

#### 2.3.9. ^1^H Nuclear Magnetic Resonance

Proton nuclear magnetic resonance (^1^H NMR) spectra of the prepared SDs were acquired using an Agilent 400 MHz digital Fourier-transform nuclear magnetic resonance (FT-NMR) spectrophotometer (Santa Clara, CA, USA) operating at a 1 H frequency of 400 MHz. Chemical shifts were recorded for the external tetramethylsilane at 0 ppm to calibrate the instrument by solvent signals (dimethylsulfoxide at 2.5 ppm, deuterated water at 4.75 ppm, and chloroform CHCl_3_ at 7.25 ppm). The spectra of samples were acquired in DMSO at 399 K.

#### 2.3.10. Nuclear Overhauser Enhancement Spectroscopy

Nuclear Overhauser effect spectroscopy (2 H NOESY) spectra of the prepared SDs were acquired using an Agilent 400 MHz digital Fourier-transform nuclear magnetic resonance (FT-NMR) spectrophotometer (Santa Clara, CA, USA), operating at a 1 H frequency of 400 MHz. Chemical shifts were recorded for the external tetramethylsilane at 0 ppm to calibrate via solvent signals (dimethylsulfoxide at 2.5 ppm, deuterated water at 4.75 ppm, and chloroform at 7.25 ppm). The spectra of samples were acquired in DMSO at 399 K.

#### 2.3.11. In Vitro Release Study

In vitro release studies on CVL and the developed SDs were carried out using the United States Pharmacopeia (USP) dissolution testing type-2 apparatus (USP Dissolution Test Apparatus, Electrolab, Mumbai). The dissolution test was performed using 900 mL of simulated gastric fluid (pH 1.6) and 500 mL of simulated intestinal fluid (pH 6.8), under non-sink conditions at 37 ± 0.5 °C and 100 rpm for 2 h and 4 h, respectively. At predetermined time intervals, samples were withdrawn, filtered using a 0.22μ membrane filter (MF-Millipore™, Merk, Darmstadt, Germany), and the amount of CVL was quantified using high-performance liquid chromatography (HPLC) with an HP1100 series equipped with a G1315A DAD detector (Agilent Technologies, Wilmington, NC, USA) using a symmetry C18 column (5.0 μm, 4.6 × 250 mm). The mobile phase consisted of methanol, 0.33 N phosphate buffer (4.5 g KH_2_PO_4_ and 0.61 g K_2_HPO_4_, dissolved in 1000 mL of purified water), and glacial acetic acid at a ratio of 60:40:0.3 (by volume); the flow rate was 1 mL/min. CVL was detected at 284 nm, with a retention time of 4.7 min. The volume of solution injected was 10 μL. The linearity of the HPLC method was noted in a concentration range of 2 µg/mL to 70 µg/mL, with a correlation coefficient of 1.000 in terms of linear regression. The concentration of CVL was quantified using the peak area method [50].

### 2.4. Infused Precipitation Study of Solid Dispersion

An infused precipitation study was carried out for the SDs, in which the SDs were dissolved in fasted state simulated gastric fluid (FaSSGF) of 0.01 N HCl with a pH of 1.2. Since CVL is hydrophobic in nature (molecular weight of 406.5 g/mol, pKa 7.8), a 0.2 mg/mL concentration of the infused solution (clinical dose 3.125 mg) was prepared. The drug solution was then infused with fasted state simulated intestinal fluid (FaSSIF) with a pH of 6.8 [51]. The flasks were gently shaken (10–25 rotations per minute), then, at predetermined time intervals, aliquots were withdrawn and filtered through a 0.22 μ membrane filter (MF-Millipore™, Merk, Darmstadt, Germany) and then analyzed. The infusion was terminated at 240 min. The CVL’s dissolving was determined by high-performance liquid chromatography (HPLC), adopting the analytical parameters and method mentioned in the previous section. The pH, particle size, and viscosity of the samples were measured. The solid form of precipitated particles during the infusion experiment was observed using a polarized-light microscope (PLM).

### 2.5. Dielectric Analysis

Dielectric analysis (DEA) was performed for the SDs by preparing pellets and subjecting them to a pressure of 2000 lbs/in^2^ by means of a hydraulic press (Model SL-89, Spectra Lab, Mumbai, India) for a few minutes. The obtained pellets were then used for dielectric measurements and were further analyzed, as follows.

#### 2.5.1. Frequency Domain Spectroscopy

Dielectric characterization was performed using a precise high frequency inductance capacitance resistance (HF LCR) meter from 20 Hz to 10 MHz, using a Wayne Kerr 6510P meter (Wayne Kerr Electronics, West Sussex, UK). The capacitance (C), dissipation factor (tan δ), and conductance (G) were measured over set temperature conditions (25 °C) by a varying frequency range, from 150 Hz to 700 kHz, at an applied voltage of 1 V (rms), as per the ASTM D150-98 standard.

The dielectric constant (ε′) (real part) was calculated from the measured capacitance, using the equation given below:

$$C=\frac{\varepsilon o\;\varepsilon^{\prime }A}{t}$$

where C is the capacitance in F, εo is the absolute permittivity (εo = 8.854 × 10–13 F/m), A is the area of the electrode (in m^2^), and t is the thickness of the specimen (in m).

The dielectric loss (ε″) (imaginary part) was calculated from the measured tan δ, using the equation: ε″ = ε′ tan δ.

The AC conductivity (σac) was computed using the relationship: σac = Gt /A, where G is the measured conductance in Siemens, t is the thickness of the sample (m), and A is the area of the electrode (m^2^).

#### 2.5.2. Polarization and Depolarization Current Measurements

The polarization and depolarization currents (PDC) were measured with a PDC analyzer (DIRANA, model EN 50110-1). Initially, the samples were charged for 100 s, with a DC voltage of 200 V, then the voltage was decreased. The discharged currents, through a time period of charging, discharging, and current measurement, were all electronically controlled and recorded through the inbuilt software feature. The DC conductivity of the test sample was estimated using the following equation:

$$\sigma =\frac{\varepsilon r}{CoUo}[i_{p}(t)-i_{d}(t)]$$

where σ is the conductivity of the composite, ε r is the relative permittivity, Co is the geometric capacitance, Uo is the step voltage, i_p_(t) is the polarization current, and i_d_(t) is the depolarization current.

#### 2.5.3. Electrical Resistivity

The volume and surface resistances of the drug and SDs were measured experimentally using a megohmmeter (Sefelec M1501M, Sefelec SAS, Lognes, France), in accordance with ASTM D 527-91. The volume resistivity (ρv) was calculated using the following equation:

$$\rho_{v}=\;\frac{\;\mathrm{Rv}\;A}{t}$$

where A is the area of the measuring electrode, Rv is the volume resistance, and t is the thickness of the sample. The surface resistivity, ρv, of the sample was calculated using the equation ρs = Rs X perimeter of the electrode/guard gap, where Rs is the surface resistance of the API, and the solid dispersion at the perimeter = π D, where D is the diameter of the electrode. The surface resistivity (ρs) = Rs X perimeter/g, where Rs is the surface resistance (Ω), and perimeter = π D, where D is the diameter of the sample and g is the guard gap [52,53,54].

### 2.6. In Vivo Analysis

#### 2.6.1. Oral Pharmacokinetic Studies

The pharmacokinetic studies were performed on healthy Swiss albino Wistar rats (8–9 weeks, 200–300 gm) to evaluate the oral bioavailability of the drugs from SDs. All animal experiments were approved by the Institutional Animal Ethics Committee (approval no. JSSCPM/IAEC/283/2018). The rats were housed in controlled environmental conditions at a temperature of 25 ± 2 °C and relative humidity (RH, 55 ± 5%); the rats were acclimatized for 7 days under standard laboratory conditions before starting the experiment. The animals were kept in polypropylene cages, with free access to a standard laboratory diet and water. The rats were fasted overnight, with free access to water. SDs at a dose of 20 mg/kg body weight were orally administered to three groups of albino rats. Blood samples (0.3 mL) were collected from the tail vein in EDTA-coated Eppendorf tubes at 0.5, 1, 2, 4, 6, 8, 12, 16, and 24 h after drug administration. All the blood samples were centrifuged (5000 rpm, 15 min) and plasma was collected and stored at −80 °C until further analysis. Pharmacokinetic data analysis was carried out using pharmacokinetic software (PharmPK, PCModfir, Version 6, free trial). The concentrations of the drug in plasma were determined using a validated HPLC method as quoted in the aforementioned Section 2.3.11. An analysis of variance (ANOVA) test was performed to analyze the differences between the groups; the results were further verified by Tukey’s multiple comparison test, using GraphPad Prism 8.0.2 software (GraphPad Software Inc., San Diego, CA, USA).

#### 2.6.2. In Vivo Irritation Test

The in vivo irritation study was conducted on albino Wistar rats to estimate the samples’ tolerance in the gastrointestinal tract. The rats (weighing 300 ± 20 gm) were randomly divided into three groups, comprising three animals in each group, with free access to food and water. SDs and CVL (10 mg/kg body weight) were orally administered to rats in all groups once a day for 7 days, respectively. First, 1 mL in volume of the test/standard solution was given by the oral route; the control group only received the same amount of saline once a day for 7 days, orally. After the 7th day of dosing, 2 h after the last administration, the rats were euthanized, and the tissue samples were collected from the intestine (a 5-mm length of the small intestine). Subsequently, the samples were fixed with 4% formaldehyde, embedded in paraffin, and sliced into histological sections for histopathology assessment. Sections of tissue were cut using a microtome and stained with hematoxylin and eosin (H&E) before microscopic examination. In this investigation, gastric mucosal irritation was studied in Wistar albino rats after the oral administration of SDs, in comparison with CVL [55,56].

### 2.7. Statistical Analysis

All the recorded research data, wherever mentioned, were statistically analyzed using the GraphPad Prism 8.0.2 software (GraphPad Software Inc., San Diego, CA, USA). The results are mostly expressed as the mean ± SD for (*n* = 3 to 15) independent experiments. A two-way ANOVA with a post hoc analysis implementing Tukey’s multiple comparisons test was used to assess the differences among the groups. *p* < 0.05 was considered to be a statistically significant difference among all the groups (*: *p* < 0.05, **: *p* < 0.01, ***: *p* < 0.001, and ****: *p* < 0.0001 (in comparison to the control group)).

## 3. Results and Discussion

### 3.1. *Inhibition Effect of Polymers by Recrystallization*

**Figure 1:**
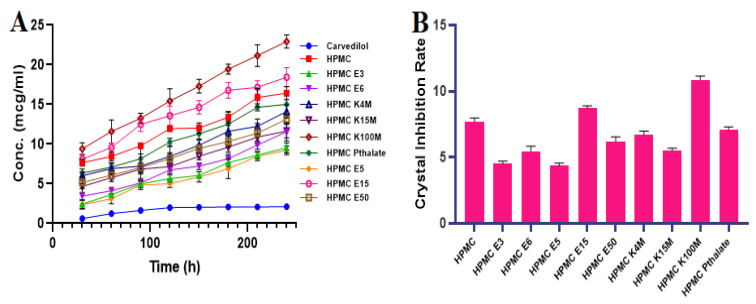
(**A**) The inhibition effect and (**B**) the crystal growth inhibition rate of the different grades of cellulose polymer.

The inhibition effect of cellulose polymer and its different grades (Table 1) and the supersaturated state of CVL were analyzed in fasted state of simulated intestinal fluid (Figure 1A,B). CVL, in its saturated state, tends to precipitate quickly from the test medium, leading to low concentrations (3.1 µg/mL) at 240 min. The presence of polymers successfully inhibited the nucleation and crystal growth of CVL in the concentrated solution in the supersaturated state. Among all the HPMC polymers, HPMC K100M showed good results in terms of inhibiting nucleation and crystal growth, while HPMC E5 exhibited the least inhibition effect. Among the different grades of cellulose polymers, the ranking order of inhibiting crystallization from the strongest to weakest was HPMC K100M > HPMC E15 > HPMC > HPMC phthalate > HPMC K4M > HPMC E50 > HPMC K15M > HPMC E6 > HPMC E3 > HPMC E5. This cellulose polymer, with a high molecular weight and higher viscosity grades of HPMC, influences the nucleation inhibition more prominently than the lower molecular weight and higher viscosity grades. This could be attributed to a greater number of -NH groups and a greater number of -OH groups in the drug, leading to intermolecular interactions between the drug and the polymer. The outcomes of the influence of cellulosic polymer viscosity on crystallization inhibition showed that the higher viscosity grades (HPMC K100M) are comparatively more effective in inhibiting the crystallization of CVL in its supersaturated state, whereas HPMC E5 exhibited the least crystallization inhibition potency. The cellulosic polymers exhibited evident potential, with a strong impact on crystallization inhibition, suggesting that cellulose polymers are a good choice and can serve as polymeric precipitation inhibitors. The change in the molecular weight of the cellulose polymers would significantly affect the rate of absorption of CVL in a supersaturated state. The inhibitory action against CVL crystallization in the presence and absence of the polymer may be due to hydrogen bonding between the drug and the polymer molecule, which could be affected by the presence of a number of hydroxyl groups per certain weight of cellulose polymer.

### 3.2. *Formulation and Evaluation of CVL-SDs*

#### Preparation of the Solid Dispersion

The “hit polymers” (HPMC K100M and HPMC E15LV), obtained from inhibiting the recrystallization of CVL in the supersaturated state, were formulated into CVL-SDs. The prepared SD-obtained practical yield was found to be 96.95 ± 1.13%, and the drug content was found to be 98.67 ± 1.06% for HPMC K100M (SDA/KM), while for HPMC, the E15LV (SDC/ELV) practical yield was found to be 82.71%, and the drug content was found to be 83.12%. The main purpose of the present work was to achieve the ASDs of CVL (dispersed in the polymer matrix) which in turn, has allowed the ability to generate and maintain supersaturation. These values are within acceptable ranges, indicating the minimal loss of the drug during the process of the solvent evaporation method. After this, the prepared CVL-SDs were evaluated and characterized. Nevertheless, the exact mechanism which leads to the release enhancement was thoroughly investigated for the qualitative evaluation of interactions.

### 3.3. Characterization of CVL-SDs

#### 3.3.1. Powder X-ray Diffraction

**Figure 2:**
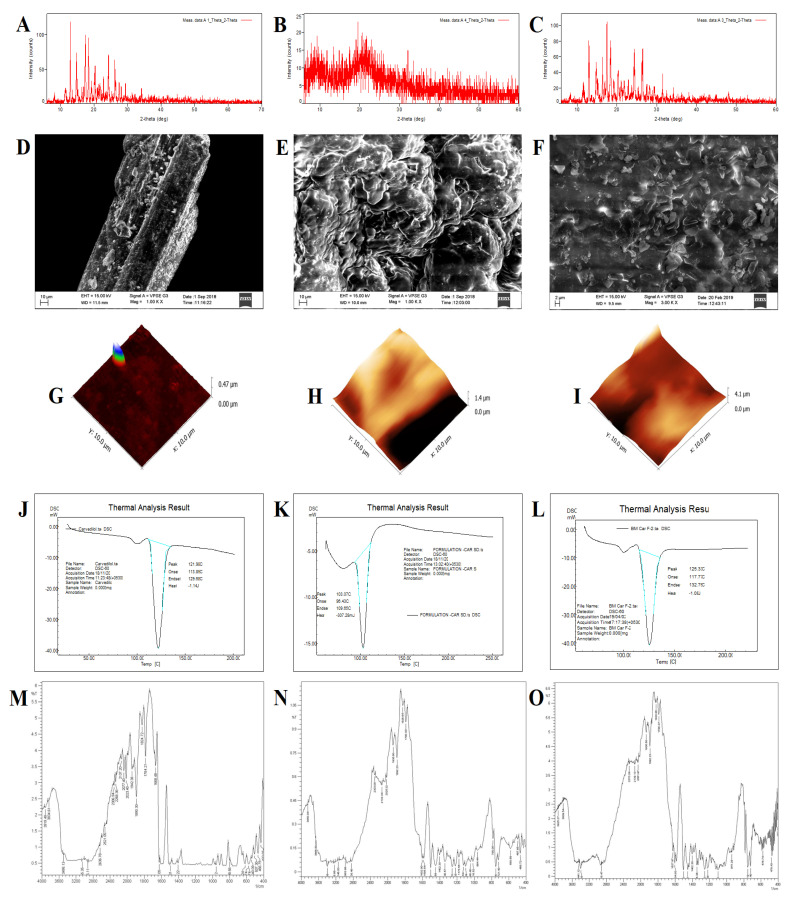
Evaluation results noted for CVL and CVL-SDs. Powder X-ray diffraction. (**A**): CVL, (**B**): CVL-SDA/KM, (**C**): CVL-SDC/ELV; scanning electron microscopy—(**D**): CVL, (**E**): CVL-SDA/KM, (**F**): CVL-SDC/ELV; atomic force microscopy—(**G**): CVL, (**H**): CVL-SDA/KM, (**I**): CVL-SDC/ELV; differential scanning calorimetry—(**J**): CVL, (**K**): CVL-SDA/KM, (**L**): CVL-SDC/ELV; FT-IR spectroscopy—(**M**): CVL, (**N**): CVL-SDA/KM, (**O**): CVL-SDC/ELV.

Powder X-ray diffractograms have helped in understanding the physical characteristics and purity for differentiating CVL-SDs (Figure 2). The crystalline nature of pure CVL was revealed in the characteristic sharp diffraction peaks at 2θ 12.8°, 15.62°, 17.46°, 18.56°, 20.1°, 24.3°, and 26.2° (Figure 2A). The CVL-SD SDA/KM depicted a reduction in peak intensity, with minor diffraction peaks at 12.8° and 15.62° indicating a decrease in the crystallinity of CVL in the prepared formulation, which comprises a stable ASD. The transformation to an amorphous nature could be attributed to the strong interactions between the drug and polymer (devoid of any phase separation) during the SDs preparation using the solvent evaporation method (Figure 2B). These results suggest no crystallinity in the formulation or transformation of CVL into a crystalline phase within the polymeric matrix. Conversely, the CVL-SD SDC/ELV diffractogram showed high-intensity diffraction peaks, suggesting the crystalline nature of the formulation. Sharp diffraction peaks were noted at 2θ 26.61°, 30.31°, 36.1°, 34.1° and 22.1°, indicating the crystalline nature of the prepared SDs (Figure 2C). Collectively, all these results suggest that the crystalline nature is due to the presence of polymer HPMC E15LV and its intermolecular interactions (bonding between the individual chains of monomer), leading to aggregation and a high degree of crystallinity.

#### 3.3.2. Scanning Electron Microscopy

The SEM images of CVL appeared to show small columnar crystals (Figure 2D). The SDA/KM SDs images revealed a good effect on forming particles, as the small portion of polymer in solution aggregated into a fiber-like structure as it solidified, prior to forming particles; this facade forms particles of regular size, of 450–500 nm, indicating the evidence of ASDs (Figure 2E). The drug-polymer propelled the solvent evaporation method, which is adopted and resulted in producing a smooth surface due to the fast diffusion rate of the solvent. The surface morphology of the SDC/ELV batch revealed that the saturated solution of CVL produced rough and high-yield crystals (Figure 2F). Herein, the undissolved polymer produced irregular and rod-shaped particles. During the preparation process, the solvent diffused faster into the aqueous phase before the even development of stable crystals, causing the aggregation of irregular crystal forms.

#### 3.3.3. Atomic Force Microscopy

The AFM images revealed the surface roughness and morphology of the sample surface in three-dimensional detail, down to the nanoscale. The CVL AFM images captured revealed nanometer-sized particles with a thickness of about 0.47 µm (Figure 2G). The CVL was found to have definite height, stiffness, adhesion force, friction, and a rough surface on the fracture surface; all of these characteristics prove and establish the crystalline nature of CVL. The AFM images of SDs depicted a thickness of 1.4 µm and indicated that the formulation was molecularly homogenous on a nanometer scale, while the surface morphology was quite different from that of the native CVL, with a smooth surface, due to the smoothing effect of polymers present in the SDs (Figure 2H,I). The CVL SDs thickness of 1.4 µm (with weak drug–polymer interaction) and higher molecular mobility at high drug concentrations in saturated states can lead to faster phase separation, drug nucleation, and crystal growth. For avoiding all these aforementioned phases, the optimization and formulation of stable CVL SDs using a suitable polymer is crucial and has been developed in the present research.

#### 3.3.4. Differential Scanning Calorimetry

The DSC thermogram of CVL exhibited a sharp endothermic peak at 121.98 °C (Figure 2J). The DSC thermogram of CVL-SDs SDA-KM exhibited a sharp endothermic peak at Tpeak = 103.07 °C corresponding to its melting point (Figure 2K). This indicates that the CVL-SD was more amorphous in nature with respect to the native crystalline form of CVL. Briefly, the different thermal behavior is seen, due to the cellulose ethers, part of the hydrogen atoms of the three hydroxyl groups on the anhydroglucose-repeating unit, which is replaced by the alkyl or mixed alkyl groups. Such modifications of cellulose via esterification and etherification of the hydroxyl groups are termed cellulosic reactions. The DSC thermogram with high Tg polymers, such as HPMC, increases the wettability and hydrophilicity of the system and maintains the molecules in the disordered state for a longer period. Conversely, the DSC curve of CVL-SDs SDC/ELV showed the Tpeak = 110.82 °C, indicating a sharp endothermic peak; this suggests a crystalline nature, due to the presence of polymer (Figure 2L). Herein, the presence of polymers results in weak intramolecular and intermolecular hydrogen bonding between the chains and results in a high degree of crystallinity.

#### 3.3.5. Fourier-Transform Infrared Spectroscopy

The FT-IR spectrum of the pure drug CVL showed characteristic peaks (Figure 2M) at 3346.27 cm^−1^ (O-H and N-H stretching vibration peaks, merged together), 2925.81 cm^−1^ (C-H stretching vibrations), 1598.88 cm^−1^ (N-H bending vibrations) and 1253.64 cm^−1^ (O-H bending and C-O stretching vibrations). The distinct peaks observed in the FT-IR spectrum of SDA/KM were 3850 cm^−1^ (OH stretching), 2916 cm^−1^ (CH stretching), 1602 cm^−1^ (CO/CC stretching), 1585 cm^−1^ (C=C stretching), 1446 cm^−1^ (CN stretching), 1256 cm^−1^ (CC stretching), 1215 cm^−1^ (C-CH_3_, CC stretching), 1045 cm^−1^ (CH_3_ rocking), 805 cm^−1^ (CCC ring bending), 785 cm^−1^ (CCC in-plane bending), 721 cm^−1^ (C-OH bending) and 620 cm^−1^ (CCC out-of-plane bending) (Figure 2N). The shifting of distinct peaks was observed in the FT-IR spectrum for the SDC/ELV as 3925 cm^−1^ (OH stretching), 3321 cm^−1^ (NH stretching), 2270 cm^−1^ (CH stretching), 1627 cm^−1^ (CO stretching), 1504 cm^−1^ (C=C stretching), 1423 cm^−1^ (CN stretching), 1178 cm^−1^ (CH in-plane bending), 770 cm^−1^ (CH_3_ wagging) and 620 cm^−1^ (CCO in-plane bending) (Figure 2O). The disappearance of peaks was observed at around 800–620 cm^−1^ and the changes in the FT-IR spectra suggest a weak van der Waals interaction among CVL and HPMC E15LV, resulting in a stable crystalline complex.

#### 3.3.6. Raman Spectroscopy

**Figure 3:**
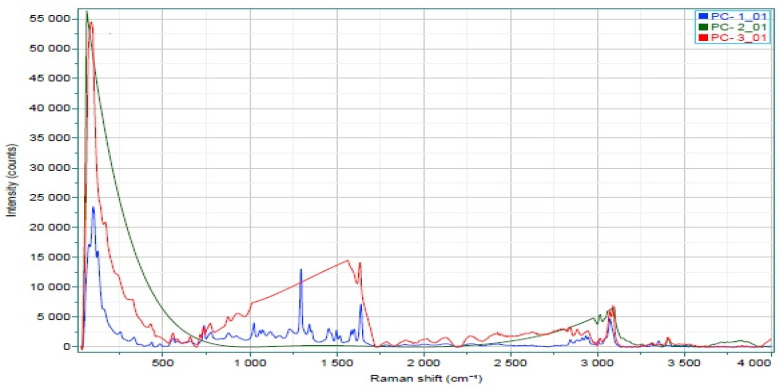
Raman spectroscopy analysis of CVL (Blue color), CVL-SDs (SDA/KM -Green color; SDC/ELV-Red color).

Raman spectroscopy is a useful tool for the characterization of molecular interactions between drugs and polymers in SDs. In Raman spectroscopy, less intensely scattering light is measured by the detector; this is called Stokes scattering. Stokes scattering is generally used in the pharmaceutics field because most drugs and excipients are in the ground state. Raman spectroscopy was employed to identify the molecular interaction between CVL and a prepared SD of polymers (Figure 3). CVL has a peak of free form at 89.38 cm^−1^ a stable form of crystal lattice, and acyclic vibrations (-C-C). Aliphatic chain vibrations were present at 1294 cm^−1^; the 1647.15 cm^−1^ peak indicates moderate to weak vibrations (-C=C), while the peak at 3073.09 cm^−1^ corresponds to the (-OH) of the pure drug, and the peak at 3401.15 cm^−1^ corresponds to the presence of the (-NH) group. The peak observed for SDA/KM at 89.38 cm^−1^, corresponding to a stable form of crystal lattice, was maintained throughout the formulation and, in the pure drug, as the free form (indicating good stability). The peak at 1294 cm^−1^ was lost due to structural rigidity, while the peak at 1647.15 cm^−1^ indicates the (-C=C) vibration disappearance in the formulation, either due to lost or rigidified pi-electron movements. Peaks at 3073.09 cm^−1^ and 3401.15 cm^−1^ indicated the formation of weak (-OH) and (-NH) bonds, respectively. Furthermore, the flat peak indicates that the drug’s structural flexibility has become rigid and that the drug has been encapsulated well in the polymer. Conversely, the CVL-SDs SDA/ELV spectrum has a peak at 92.50 cm^−1^, suggesting that the drug crystal lattice was maintained in SDs. A characteristic peak at 1561.05 cm^−1^ corresponding to the C=C vibrations was present in the pure drug as well as in the SDs, with the polymer HPMC E15LV showing a pi-electron movement. In SDs, both the (-OH) and (-NH) bonds were present but had weak bonding; thus, this finding leads to the structure being less rigid when compared with the polymer, HPMC K100M.

#### 3.3.7. Dynamic Light Scattering

**Figure 4:**
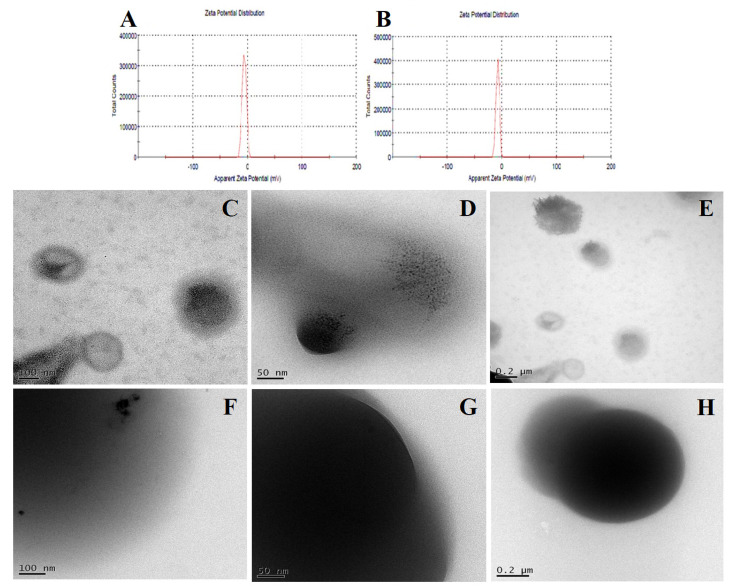
DLS size-distribution plot and cryo-TEM images of the SDA/KM (**A**,**C**–**E**) and SDC/ELV (**B**,**F**–**H**).

To better understand the mechanism of polymer-mediated drug supersaturation, the DLS technique was employed, which characterizes any sub-visible drug-polymer aggregates in the clear supernatant obtained from the supersaturation study. The SDA/KM with polymers exhibited a strong negative zeta potential of −6.16 mV (Figure 4A), which significantly helps to stabilize the SDs, formed with good hydrogen bonding or hydrophobic interaction between the SD moieties. The particle size of the SDA/KM was quite different, with a mean diameter of 423–450 nm (PDI 0.169) and a zeta potential of +3.80 mV. For SDC/ELV, a negative zeta potential of −7.91 mV was noted (Figure 4B); this might have been the result of low molecular interaction when compared to ASDs. The SDC/ELV showed a particle size of 1220–1250 nm (PDI 0.327) and exhibited a zeta potential of +2.91 mV in the aqueous environment, which results in the formation of sub-visible aggregates, subsequently helping in maintaining the supersaturation.

#### 3.3.8. Transmission Electron Microscopy

The TEM and TEM images are useful methods in the study, producing both real-space images and electron diffraction patterns to identify the crystalline drugs in CVL-SDs [57]. The SDs of SDA/KM exhibited a spherical shape, with clear signs of erosion from the polymer matrix (Figure 4). This also suggests that CVL is embedded and uniformly distributed in the polymeric matrix and confirms the amorphous nature of CVL-SDs. Conversely, when subjected to TEM imaging, the results also revealed few instances of agglomerates of the primary nanoparticles forming secondary nanoparticles [58]. These results suggest that the drug was uniformly distributed in the polymeric matrix by transforming into a non-crystalline state and that this tends to increase the crystallinity in the formulation.

#### 3.3.9. Proton Nuclear Magnetic Resonance and NOESY

**Figure 5:**
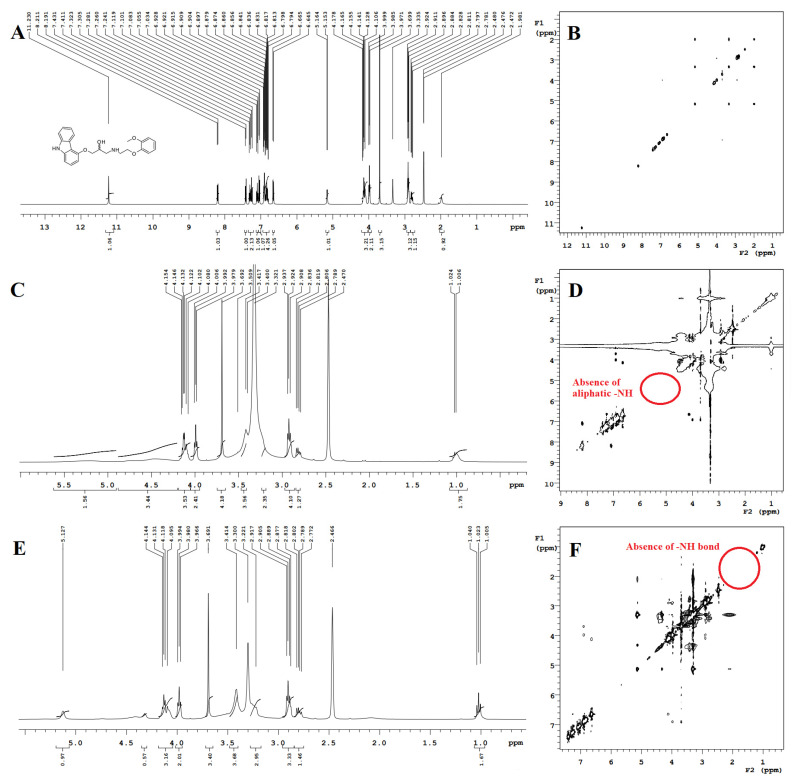
1H-NMR and NOESY images of CVL (**A**,**B**), CVL-SDA/KM (**C**,**D**), and CVL-SDC/ELV (**E**,**F**).

The pure CVL and CVL-SDs samples were subjected to ^1^H-NMR spectral analysis; the recorded spectra are presented in Figure 5. The ^1^H-NMR spectra of SDA/KM revealed the disappearance of the peak at 5 ppm; this result indicates the probable interaction between the OH group and the NH moiety. Briefly, the hydroxyl group (-OH) of HPMC K100M has interacted with the hydroxyl group (-OH) of CVL. This interaction, where the -OH from the polymer and the -OH of CVL are the hydrogen bond donor and the hydrogen bond acceptor, respectively, results in the disappearance of a specific -OH peak at 5.2 ppm. Conversely, the ^1^H-NMR spectra of SDC/ELV showed the disappearance of the peak at 2 ppm, indicating a probable interaction between the -OH group and the NH moiety. Furthermore, the hydroxyl group (-OH) of HPMC E15LV interacted with the aliphatic amine group (-NH) of CVL, wherein the -OH group from the polymer and the -NH group from the drug behaved as the hydrogen bond donor and hydrogen bond acceptor, respectively. These interactions have resulted in the disappearance of a specific aliphatic -NH peak at 2 ppm. Additionally, the ^1^H-NMR spectra of CVL-SDs exhibited the NH peak, but the disappearance in the spectrum of SDC/ELV confirmed that in the CVL-SDs, the NH groups were connected with the -OH groups. Drug-polymer interactions critically impact the initial dissolution as well as the drug supersaturation and, eventually, the oral bioavailability of ASDs. Furthermore, the NOESY spectra also clearly indicated that the adjacent hydrogen atoms present in the structure of CVL were retained in the SDs. All these results of the ^1^H-NMR spectral analysis and NOESY analysis have confirmed the molecular interactions between the drug and polymer during the formulation of ASDs, without hindering the drug’s efficiency and stability.

#### 3.3.10. In Vitro Release Study

**Figure 6:**
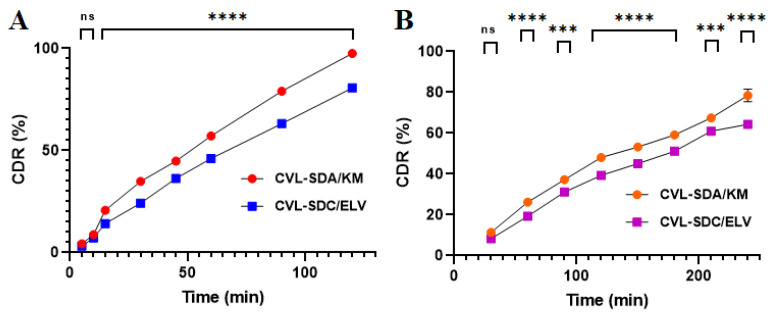
In vitro release profiles of CVL-SDs in (**A**) FaSSGF pH 1.2 and (**B**) FaSSIF pH 6.8 (data expressed as the mean ± SD, *n* = 3; ns—non-significant, * *p* < 0.05, ** *p* < 0.01, *** *p* < 0.001, **** *p* < 0.0001).

The in vitro release study of the CVL-SDs was conducted in simulated fluids (pH 1.2 and 6.8) to demonstrate the controlled release of SDs. Figure 6 shows the in vitro release profiles of CVL-SDs in different dissolution media, i.e., simulated gastric fluid (FaSSGF, pH 1.2) for the first 2 h, followed by simulated intestinal fluid (FaSSIF pH 6.8) for 4 h. The in vitro dissolution study was conducted under non-sink conditions to better predict the in vivo performance of the precipitation inhibitors, with respect to the sink condition. The SDA/KM SDs exhibited a matrix diffusion-controlled release, with 97.56 ± 1.2% and 78.45 ± 3.1% of drug release in the simulated gastric and intestinal fluid, respectively. Controlled-release cellulose polymers containing the carboxyl group remained unionized at the acidic pH and prevented drug release in the gastric region. The rigid cellulosic polymer backbones act by sterically hindering re-crystallization and helping in the maintenance of supersaturation. Stable ASDs were achieved via the physicochemical properties and nature of the drug–polymer, such as a high molecular weight and resistance to hydrolysis, which can prevent the absorption of most of the cellulose ether and ethers in the GI tract. The in vitro release data was subject to adequacy calculations and was fitted to various kinetic mathematical models. The Peppas model outcomes depicted the highest value of regression coefficient (r^2^ = 0.968), which indicates a non-Fickian drug release from the polymer matrix, which swells and dissolves over time, rather than due to polymeric erosion. Conversely, CVL-SDs and SDC/ELV showed release behavior, with 80.63 ± 1.1% and 64.33 ± 1.6% of drug release in the simulated gastric and intestinal fluid, respectively. The controlled or prolonged release of CVL-SDs could be attributed to the poor intramolecular and intermolecular hydrogen bonding between the drug and polymer, leading to a high degree of crystallinity, which subsequently decreases the drug’s release due to its poor wettability and adsorption properties. These findings, together with the release kinetics, the mathematical models’ analyses, and the outcomes thereof, suggest that the mechanism of drug release from the polymeric matrix was from matrix-swelling dependent diffusion, rather than from polymeric erosion.

### 3.4. Infused Precipitation Study

An infused precipitation study was performed to examine the changes in CVL precipitation and crystal growth. The rate of infusion precipitation obtained from this study has helped to interpret and understand the effectiveness and stability of the molecular mobility, miscibility, crystallinity, and crystallization in a supersaturated state.

#### 3.4.1. Solution Phase Transfer (SPT)

**Figure 7:**
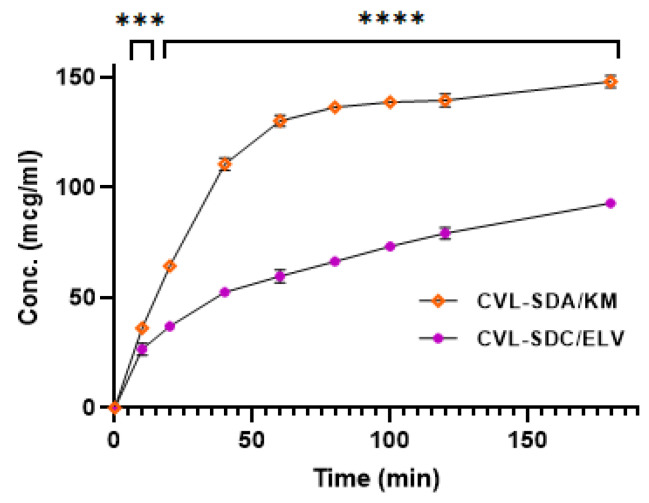
Solution phase transfer profiles of CVL-SDA/KM and CVL-SDC/ELV (data expressed as mean ± SD, *n* = 3; ns—non-significant, * *p* < 0.05, ** *p* < 0.01, *** *p* < 0.001, **** *p* < 0.0001).

During the process of infusion/solution phase transfer, pH changes occur, from pH 1.6 FaSSGF to pH 6.8 FaSSGF. During the transfer process, at first, the drug is dissolved in the acid medium, while the remaining amount of the drug infused into the pH 6.8 media rapidly crystallizes, which results in a reduced rate of absorption (Figure 7). The presence of a cellulose polymer hinders molecular aggregation, nucleation, crystal growth, and precipitation by considering the “spring and parachute” theory [59]. The high-energy supersaturated form is thermodynamically unstable and has a tendency to precipitate and crystallize; this is referred to as the “spring”. “Parachutes” maintain high concentrations and inhibit drug precipitation for an extended period of time. The polymers, which act as precipitation inhibitors, inhibit the recrystallization of CVL in its supersaturated state. It was obvious that the CVL-SDs SDA/KM exhibited controlled release and did not tend to precipitate for a longer period, in comparison to the native CVL. The cellulose polymer HPMC K100M, employed in the formulation, acts as a pH-responsive agent and depicts strong hydrogen bond donor and acceptor properties; this results in high stability and the performance of ASDs, whereas, in the case of the HPMC E15LV polymer, this formed SDs with weak intermolecular interaction among the donors and acceptors, which would eventually result in structurally less stable SDs, consequently resulting in a decreased release from SDC/ELV and leading to SDs with a crystalline structure.

#### 3.4.2. Polarized Light Microscopy (PLM)

**Figure 8:**
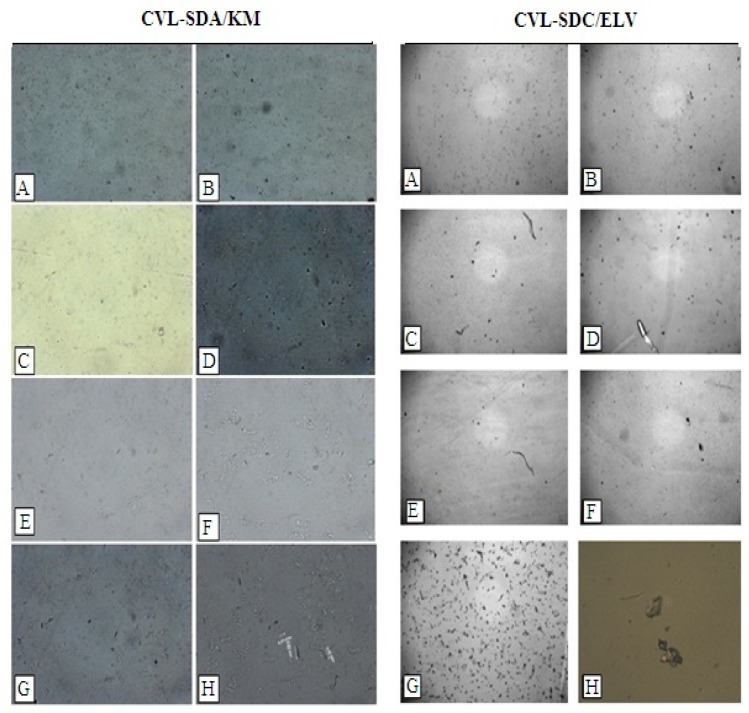
PLM images of CVL-SDs on time function (**A**) 0 min, (**B**) 20 min, (**C**) 40 min, (**D**) 60 min, (**E**) 100 min, (**F**)140 min, (**G**) 180 min and (**H**) 24 h.

The PLM images (Figure 8) have revealed notable changes in the inhibition of the crystal growth of CVL-SDs. The SDA/KM showed a marked decrease in the degree of crystallinity and continued to be in an amorphous form; this is due to the presence of a highly hydrophilic polymer with strong hydrogen-bond donor and acceptor groups. The SDA/ELV group showed an increase in crystallization tendency and continued to be in a crystalline form, due to its poor solubility in water in its native form, owing to the weak intermolecular and intramolecular hydrogen bonding that occurred between the individual chains. This enhancement could be attributed to the presence of cellulose polymers, which are inherently hydrophilic. Furthermore, a low degree of substitution of nearly any substituent, including the non-polar ones, can disrupt the H-bonding and thereby impart water solubility.

#### 3.4.3. The pH Measurement

The pH was measured using a digital pH meter (Mettler Toledo MP220, Greifensee, Switzerland) at different time intervals during the CVL-SDs-infused precipitation study. The rationale behind selecting cellulose polymers as polymeric precipitation inhibitors was to inhibit the precipitation and provide stable drug-polymer ASDs. The SDA/KM did not show any marked change in pH 6.8 over the time period; this might be because the HPMC K100M did not permit the CVL to become ionized in the acidic pH, due to the presence of a carboxyl group in the cellulose polymer that remains unionized in an acidic pH. The presence of HPMC E15LV as a precipitation inhibitor showed poor ionic strength and unfavorable charge-charge repulsion between the polymer and the drug in the solution phase, resulting in increased crystal growth.

#### 3.4.4. Viscosity and Particle Size Measurement

The viscosity measurement of the CVL-SDs was carried out with a Brookfield viscometer (DV-II, LV model, Brookfield, WI, USA), using a small volume adaptor with a thermostated water jacket and an SC4-18 spindle. The viscosity was measured (*n* = 3) at 37 °C at a rotational speed of 50 rpm, with a torque of nearly 10%. The SDA/KM did not mark any change in the viscosity and exhibited a polydispersity index of 0.169; the average particle size was found to be 1362 nm. Conversely, the SDC/ELV showed a marked increase in viscosity, with an average particle size and polydispersity index of 2305 nm and 0.506, respectively. As expected, the results suggested that the presence of the HPMC E15 LV polymer facilitates larger CVL crystal formation, which results in unstable complexation due to the higher viscosity of the system.

### 3.5. Dielectric Constant

Dielectric analysis (DEA) measures the mechanical properties of a material and is closely related to frequency-dependent dynamic mechanical analysis (DMA). The dielectric constant is a measure of the reduction of the Coulomb interaction between ion pairs in polymer electrolytes; it characterizes the ionic or molecular interactions in the electrolyte and aids in understanding the ion transport behavior.

#### 3.5.1. Frequency of Domain Spectroscopy

**Figure 9:**
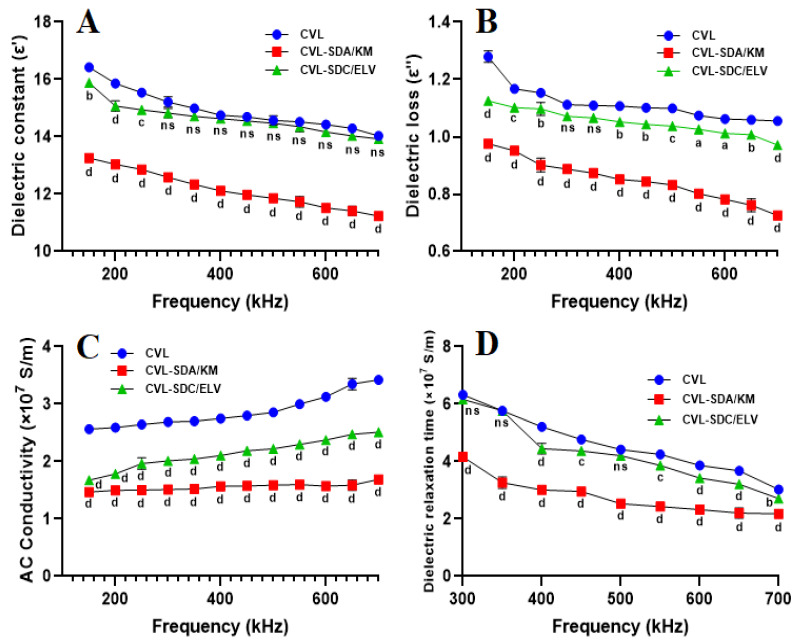
Dielectric analysis of the (**A**) dielectric constant, (**B**) dielectric loss, (**C**) AC conductivity, and (**D**) dielectric relaxation time (data expressed as mean ± SD, *n* = 3; ns: non-significant, a: *p* < 0.05, b: *p* < 0.01, c: *p* < 0.001, d: *p* < 0.0001, with respect to the control group (CVL).

The dipoles of the CVL functional group can orient themselves easily at low frequencies of the applied voltage, resulting in an increase in permittivity because of the increase in polarization (Figure 9). As the frequency changes, some of the hydroxy propyl dipole groups cannot keep pace with the applied field. This leads to the reduced contribution of dipole groups to the dielectric constant, which tends to show a decrease in the dielectric constant of CVL-SDs. The dielectric constant ε′ of SDA/KM was noted to be higher than SDC/ELV at 25 °C and at set frequencies. This is because of the fact that the dielectric permittivity of the polymeric material depends on the polarizability of the molecules; the higher the polarizability of the molecules, the higher the permittivity of the material. The dielectric loss decreases with an increase in frequency and also increases for every frequency with an increase in temperature. The dielectric loss depends on the electrical conductivity of the ingredients/materials; this is again dependent on the amount of charge carried by the ingredients and materials. The addition of polymers involves a large volume fraction at the interface and entanglements, and the motion of the charged carriers is inhibited. The lower dielectric loss in the SDA/KM matrix could be attributed to the inclusion of an HPMC K100M cellulose polymer, which balances changes in the interaction zone and manages the electrical charges better.

#### 3.5.2. The Relationship between Conduction Current and Crystallinity

The AC conductivity of the material was measured using a simple relationship between AC conductivity at a constant temperature of CVL-SDs. The behavior of ASDs exhibited flat lines, showing no interaction in conductivity, while the crystalline solid dispersion exhibited a drastic increase in conductivity. Conversely, an increase in conductivity was noticed for SC/ELV, due to the formation of crystalline SDs; being in the polymer matrix increases the drug charge and, as a result of the heat treatment, conductivity increases due to the increased mobility of ions in the drug-polymer.

#### 3.5.3. Electrical Resistivity

Dielectric relaxation is the exponential decay of polarization over time, when an externally applied electrical field is removed. The time of polarization is reduced to 1/e times by its original value, e being the natural logarithmic base, defined as the relaxation time. The dielectric relaxation (τ) of CVL-SDs (the plot of dielectric relaxation time) with respect to the frequency at 25 °C, depicted a decrease, with an increase in frequency. This relationship that was observed between AC conductivity and frequency for CVL-SDs indicates non-Arrhenius behavior, i.e., the temperature dependence of ionic conductivity.

### 3.6. In Vivo Analysis

#### 3.6.1. Oral Pharmacokinetic Studies

Pharmacokinetic studies were performed on Wistar albino rats, to assess the absorption efficiency of CVL-SDs. In comparison to CVL, SDA/KM and SDC/ELV exhibited higher C_max_ (2.01- and 1.36-fold), AUC_0–t_ (2.04- and 1.41-fold), and T_max_ (≈2-fold) values (all *p* < 0.05). These findings were in accordance with the results recorded from the in vitro dissolution, indicating that the differences in CVL-SDs absorption are primarily due to the dissolution and intestinal absorption behavior of CVL with the varied particle sizes (Table 2). The absorption of drugs from the gastrointestinal tract (GIT) involves several unit processes, including the disintegration of the dosage form, the dissolution of the drug in the gastrointestinal fluids, and the adsorption and complex binding in the GI fluids for absorption across the epithelial cells of the intestinal wall. The uptake mechanism of the CVL-SDs may involve uptake and absorption, mediated via the M-cells in the Peyer’s patches of the GIT. Furthermore, the variations in absorption could also be attributed to the increased aqueous solubility of CVL and the size effect of the CVL.

#### 3.6.2. In Vivo Irritation Test

**Figure 10:**
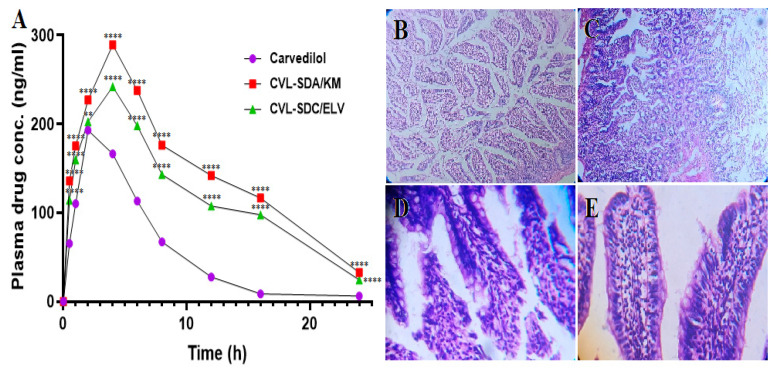
The oral kinetics profiles of the CVL-SDs (**A**) and the histopathological images of the rat duodenum cross-section of the control group (**B**), CVL group (**C**), SDA/KM group (**D**), and SDC/ELV group (**E**). (Data expressed as mean ± SD, *n* = 3; ns—non-significant, * *p* < 0.05, ** *p* < 0.01, *** *p* < 0.001, **** *p* < 0.0001.).

In this investigation, long-term gastrointestinal irritation was assessed to establish the biocompatibility and tolerability of the developed formulation. Gastric mucosal irritation was studied in Wistar albino rats, post-oral administration of CVL-SDs, in comparison to CVL. As shown in Figure 10, the histopathology of the rat gastric mucosa treated with the CVL-SDs formulation was observed to establish the effect of the formulations on tissue integrity and cell structure. The histopathology of the rat gastric mucosa demonstrated that there were hardly any hemorrhagic mucosal erosions or disruption of the intestinal villi post-treatment with CVL-SDs SDA/KM (Figure 10D). These results indicated that there was good biocompatibility between the ASD formulation and the rat gastric mucosa. Conversely, the histopathology of the rat gastric mucosa post-treatment with CVL-SDs SDC/ELV showed signs of hemorrhagic mucosal erosions and disruption of the intestinal villi (Figure 10E). These results indicated that the CVL tends to precipitate and form crystals in the gastric mucosa and that this leads to anomalies. The SDC/ELV treatment group’s histopathology images indicated that there was an incompatibility between the preparations and that the formulations tend to precipitate and form crystals in the gastrointestinal tract, leading to irritation of the gastric mucosa.

## 4. Summary and Conclusions

In this study, CVL solid dispersions were prepared with the incorporation of a cellulose polymer by the solvent evaporation method, to achieve amorphous solid dispersions (ASDs). CVL tends to precipitate in the gastrointestinal region; hence, to overcome such precipitation and recrystallization, this study was carried out using cellulose polymer. The inhibition of CVL precipitation suggested the potential of using different cellulose polymers to inhibit CVL precipitation in a supersaturated state. The CVL in the supersaturated state was screened for its precipitation and recrystallization, in the presence and absence of different grades of cellulose polymer. The “hit polymers”, which could successfully inhibit the precipitation of CVL, were formulated into CVL solid dispersions. The developed solid dispersions with HPMC K100M showed a drug content of 98.67 ± 1.06% and a controlled release of 78.45 ± 3.1%. In addition, the evaluation outcomes of DSC, FT-IR, PXRD, and a dissolution study revealed the promising stability of HPMC K100 M-based ASDs. Dielectric analysis indicated a strong stable interaction between the drug and polymer. Solution-mediated phase transfer was established from the PLM images, suggesting the significant inhibition of CVL precipitation. Infused precipitation study outcomes have also demonstrated the stability and effectiveness of CVL-SDs, without any precipitation. The in vivo oral kinetic study of CVL-SDs SDA/KM revealed a remarkable CVL absorption, with C_max_ 2.01; this could be corroborated by the strong interaction between the drug and polymer, particularly between the -OH groups of polymers to the -NH group of the drug. The findings of gastrointestinal irritation studies, conducted for the long-term, have established the stability, biocompatibility, and tolerability of formulated CVL-SDs. To sum up, as anticipated, the existence of cellulose polymers as a hydrating matrix around the CVL has inhibited its precipitation in a supersaturated state and resulted in a controlled release pattern of CVL. From all the above findings, we conclude that by incorporating a polymeric precipitation inhibitor (HPMC K100M), a stable carrier for CVL was successfully developed for overcoming the drug precipitation and stability issues.

## Figures and Tables

**Table 1:** Properties of the different grades of polymers.

Polymer	Degree of Polymerization	Molecular Weight	Hydrogen Bond Acceptor	Viscosity	Hydroxy Propyl Content	Methoxyl Content
**HPMC**	35	1261.4	6	2.4	7.3	27.8
**HPMC E3**	37	10,000	6	3.2	8.8	29.4
**HPMC E5**	37	13,000	6	5	9.5	28.4
**HPMC E6**	49	10,000	6	6.2	9.1	28.7
**HPMC E15**	49	13,000	6	16.6	9.7	28.2
**HPMC E50**	75	20,000	6	48	9	28.8
**HPMC K4M**	155	41,000	6	4927	8.2	23.3
**HPMC K15M**	416	110,000	6	7382	8.6	23.1
**HPMC K100M**	530	140,000	6	113.384	10.5	22.8
**HPMC** **phthalate**	489	1425.15	9	136	8.1	24.4

**Table 2:** The in vivo pharmacokinetic parameters of CVL-SDs after oral administration (*n* = 6).

Parameters	CVL	SDA/KM	SDC/ELV
C_max_ (ng/mL)	193.07 ± 1.91	289.25 ± 2.11	242 ± 2.45
T_max_ (h)	2.48 ± 0.21	4.31 ± 0.63	4.01 ± 0.41
AUC_0-t_ (ng.h/mL)	1376.98 ± 16.32	2874.65 ± 49.53	1915.16 ± 23.82

## Data Availability

Data will be made available on request.
